# Joint effects of sleep behaviors and physical activity on brain structure and cognitive function

**DOI:** 10.1016/j.fmre.2025.03.022

**Published:** 2025-04-29

**Authors:** Yuanyuan Fang, Chang Cheng, Deng Ning, Yao Yao, Lusen Ran, Yuqin He, Hao Huang, Di Yao, Yanzhu Huang, Dengji Pan, Wei Wang, Wenhua Liu, Minghuan Wang

**Affiliations:** a Department of Neurology, Tongji Hospital, Tongji Medical College, Huazhong University of Science and Technology, Wuhan 430030, China; b Department of Hepatobiliary Surgery, Union Hospital, Tongji Medical College, Huazhong University of Science and Technology, Wuhan 430030, China; c Clinical Research Center, Tongji Hospital, Tongji Medical College, Huazhong University of Science and Technology, Wuhan 430030, China; d Hubei Key Laboratory of Neural Injury and Functional Reconstruction, Huazhong University of Science and Technology, Wuhan 430030, China

**Keywords:** Sleep behaviors, Sleep duration, Physical activity, Brain structure, Cognitive function

## Abstract

Sleep behaviors and physical activity (PA) are widely used to predict the risk of neurodegenerative disease. However, how sleep and PA interact to influence brain and cognitive health remains unknown. This study investigated independent, joint, and interacting associations of sleep behaviors and PA with brain structures and cognitive phenotypes using linear regression and generalized linear models among over 40,000 middle-aged and older participants in UK Biobank. We first found that unhealthy sleep patterns, abnormal sleep duration and high levels of PA were each associated with worse cognitive function and brain structure damage. Joint association analyses revealed that the group with long sleep duration and low levels of PA had the greatest prevalence of white matter hyperintensities (WMH; *β* = 0.131, *P*_FDR_ = 0.013), a key metric of brain structural changes. Interestingly, interaction analysis suggested that a high level of PA significantly mitigates brain damage in relation to long sleep duration (i.e., grey matter loss, *β*_moderate-long_ = -0.088, *β*_high-long_ = -0.013, *P*_interaction_ = 0.024). In terms of cognitive phenotypes, a combination of long sleep and low levels of PA was similarly associated with worse cognitive performance (i.e., symbol digit scores, *β* = -0.043, *P*_FDR_ = 0.018). Additionally, when stratified by PA levels, long sleep duration exhibited a negative impact on cognition compared to normal sleep duration among those with low PA, but this effect was smaller or insignificant for those engaging in moderate or high PA (i.e., symbol digit scores, *β*_low-long_ = -0.047, *P*_FDR_ = 0.029; *β*_moderate-long_ = -0.022, *P*_FDR_ = 0.025; *β*_high-long_ = -0.016, *P*_FDR_ = 0.239). Overall, maintaining a high PA level during late middle age could potentially mitigate the detrimental effects on brain structure associated with excessive sleep. Healthy sleep habits alongside an adequate PA level are essential for maintaining brain health during aging.

## Graphical abstract

**Figure:**
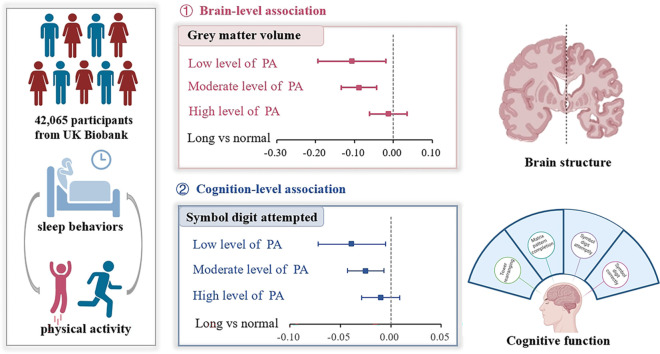

## 1 Introduction

The aging human brain shows multiple anatomic changes, including cortical and subcortical atrophy, ventricular enlargement, and alterations in white matter [[1], [2], [3]]. These structural changes have significant implications for cognitive functional status, ultimately leading to decreased independence and quality of life among older individuals [4]. As such, investigating the factors contributing to age-related changes in brain structure and cognitive function could inform specific preventive strategies for healthy aging. Such an approach could yield innovative models to integrate into public health initiatives, with particular emphasis on identifying modifiable factors that could offer relatively direct avenues for intervention.

Multiple lifestyle factors may contribute to cognitive decline among elders, particularly, sleep behavior and the level of physical activity (PA) are considered critical factors in dementia risk [5]. Previous studies have established that less PA and poor sleep quality, characterized as short/long sleep duration, difficulty falling asleep or staying asleep, or frequent disturbances, are related to worse cognitive function [5]. While a regimen of regular PA can attenuate the rate of age-related cognitive deterioration and mitigate the susceptibility to neurodegenerative disorders [6]. A considerable body of research has elucidated the positive associations between PA and the maintenance of the volume and integrity of cerebral white matter and grey matter structures [[7], [8], [9]]. Furthermore, sleep plays a pivotal role in preserving myelin plasticity and integrity [10], such that insufficient sleep duration, obstructive sleep apnea, insomnia, and excessive daytime sleepiness, are all associated with accelerated brain atrophy and white matter damage [[11], [12], [13], [14], [15]].

Sleep and PA are two time-dependent aspects of lifestyle, which may have a synergistic interaction. In a population-based study, higher PA was shown to correlate with better sleep quality [16]. Nonetheless, few studies have explored the joint associations of sleep and PA with cognitive function. To date, one cross-sectional study found the combination of poor sleep and physical inactivity worsened cognitive performance [17], and the other suggested greater levels of PA may attenuate the negative impact of poor sleep on cognition with small samples [18]. Yet, it remains unclear how PA interacts with sleep behaviors to maintain brain structure during aging. Moreover, it is still unknown whether sleep and PA have similar interactive effects on brain structure and cognition. This may call for large-scale studies designed to give a better understanding of how sleep behaviors and PA influence brain structure and cognitive aging.

To explore independent, joint, and interacted associations of sleep behaviors and PA levels with brain imaging measures and cognitive performance, we availed ourselves in this large community-dwelling population across the middle and older age in the UK Biobank. We used linear models to test our hypothesis that a certain level of PA may mitigate brain structure changes and cognitive decline arising in association with unhealthy sleep behaviors during aging.

## 2 Methods

### 2.1 Study population

**Fig. 1:**
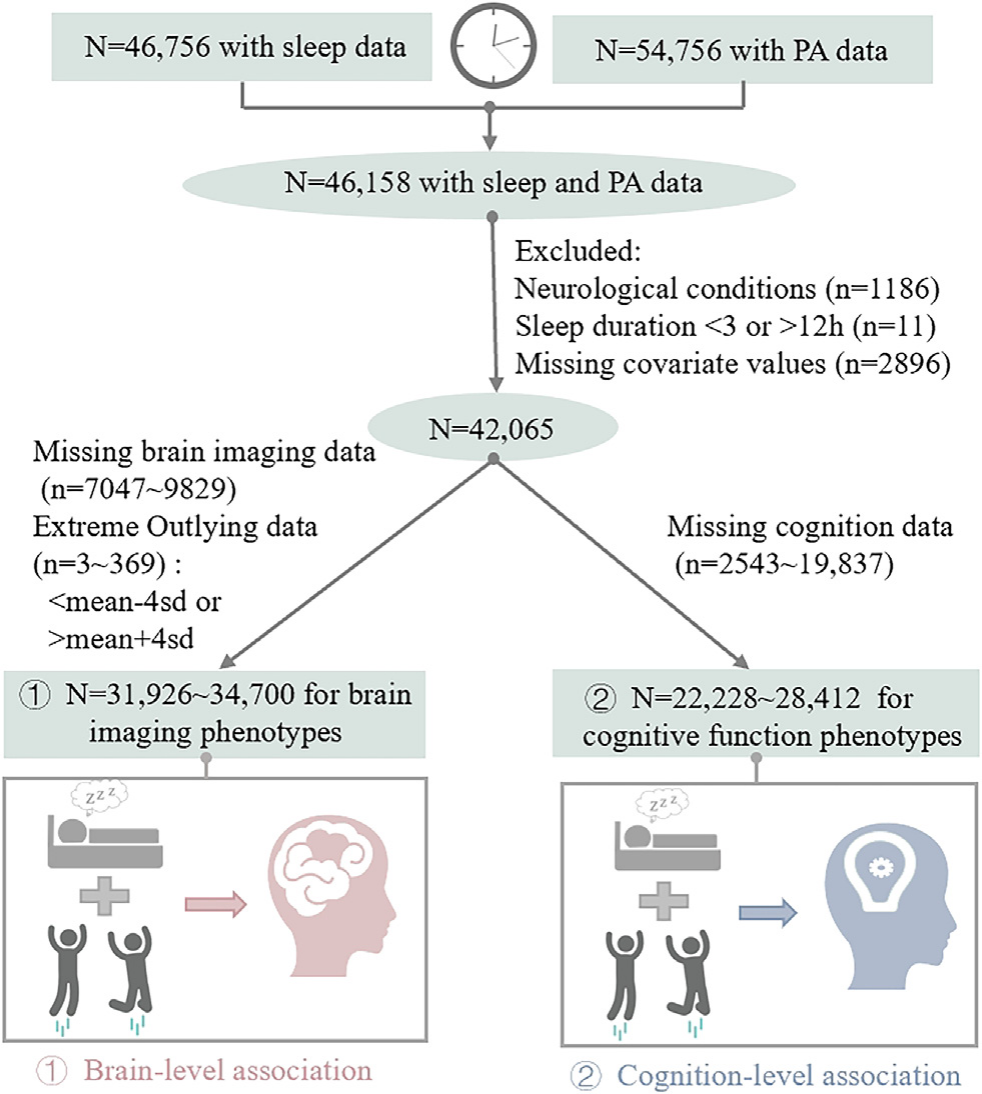
**Flow chart of the study design and sample selection**.

The UK Biobank is a population-based prospective cohort, which recruited over 500,000 community-dwelling adults aged 40–73 years across 22 assessment centers in UK between 2006 and 2010. The Biobank has recorded data in various domains, including sociodemographic, lifestyle, medical, cognitive and biological data [19]. The baseline brain magnetic resonance imaging (MRI) data was collected from 2014 to 2022, and the follow-up MRI measurements were conducted during 2019–2022. We first excluded participants without sleep and PA data and those with outlying values of sleep duration. Then, as in previous UK Biobank studies [20], we excluded individuals who reported severe neurological diseases (such as stroke, dementia, parkinsonism, and multiple sclerosis). Additional exclusion criteria included missing imaging or relevant cognitive/demographic data. Overall, the sample included 34,700 individuals for the brain imaging analyses and 28,412 participants with specific cognitive metric analyses. We additionally incorporated 3,637 participants who had two MRI measurements and no missing exposure data for longitudinal analysis. The detailed flowchart of subject selection is shown in Fig. 1. Field IDs used for our study are presented in Tables S1-S5. This research project was approved by the UK Biobank (Application Number 90,311). The UK Biobank has ethical approval from the UK National Research Ethics Service.

### 2.2 Sleep behaviors and physical activity assessment

We generated for each participant a healthy sleep score combining five aspects of sleep behavior (sleep duration, chronotype, insomnia, snoring, narcolepsy). Our definition of healthy sleep behaviors was as follows: 7–8 h sleep per day; early chronotype (‘morning’ or ‘morning rather than evening’); never or rarely insomnia; no snoring; no frequent daytime sleepiness (‘never/rarely’ or ‘sometimes’). For each aspect, the participant received a binary score of 1 if he/she classified as healthy or 0 if unhealthy, such that summation of the five components generated a composite healthy sleep score ranging from 0 to 5 [21]. We then defined the overall sleep patterns as ‘healthy sleep pattern’ (healthy sleep score ≥ 4), ‘intermediate sleep pattern’ (2 ≤ healthy sleep score ≤3), and ‘poor sleep pattern’ (healthy sleep score ≤ 1) [21].

The PA data in the UK Biobank had been obtained from a touchscreen questionnaire. First, we calculated the summed metabolic equivalent (MET) minutes per week for all activities by the reported type, duration, and frequency of PA (including walking, moderate, and vigorous PA). Then, according to the guidelines for data processing and analysis of the International Physical Activity Questionnaire (IPAQ), we categorized PA as high, moderate, and low levels (Table S3). The source data were obtained via https://biobank.ndph.ox.ac.uk/ukb/ukb/docs/ipaq_analysis.pdf.

### 2.3 MRI data management

All brain MRI data were obtained in the same 3T Siemens Skyra scanner with a standard Siemens 32-channel head coil. The sequence parameters and data processing protocols are freely available via http://www.fmrib.ox.ac.uk/ukbiobank/protocol/V4_23092014.pdf and http://biobank.ctsu.ox.ac.uk/crystal/docs/brain_mri.pdf and are also reported in previous UK Biobank studies [22,23]. In brief, T1-weighted MPRAGE and T2-weighted FLAIR volumes were acquired in sagittal orientation at 1 × 1 × 1 mm and 1.05 × 1 × 1 mm resolutions, respectively. The dMRI comprised a spin- echo-planar sequence with 10 T2-weighted (*b* ≈ 0 s mm^−2^) baseline volumes, 50 *b* = 1000 s mm^−2^ and 50 *b* = 2000 s mm^−2^ diffusion-weighted volumes, with 100 distinct diffusion-encoding directions and 2 mm isotropic voxels. The imaging-derived phenotypes, including global tissue volumes and mean white matter tract water diffusion indices, are available from the UK Biobank to approved researchers. The processing details and QC pipeline were reported in an open-access article [23]. Our outcomes of interest included grey and white matter volume (GWV), grey matter volume, peripheral cortical grey matter volume, white matter hyperintensity (WMH) volume, peri‑ventricular white matter hyperintensity (PWMH) volume, and deep white matter hyperintensity (DWMH) volume (Table S4). Extreme outlying data (outside mean ± 4 standard deviations (SD)) were excluded from the image analyses.

### 2.4 Cognitive data assessment

Cognitive testing in the UK Biobank was administered via an automated touchscreen computer at each instance, and detailed information can be found at the UK Biobank website and elsewhere [24]. In the current study, we confined the main analyses to data recorded at the initial imaging visit. A total of four cognitive phenotypes were used, including symbol digit substitution (2 measures), matrix pattern completion, and tower rearranging test. Detailed information on these phenotypes and their field IDs in the UK Biobank are provided in Table S5.

### 2.5 Covariates

The potential covariates included sex, age, ethnicity (white or other), education level (no qualification, low, medium, or high), Townsend deprivation index (TDI), smoking status (never, previous, or current), alcohol intake frequency (never, occasionally, or always), body mass index (BMI), waist-hip ratio (WHR), hypertension (yes or no), diabetes (yes or no) and lipidaemias (yes or no). For details of these demographics and covariates, see Table S1.

### 2.6 Statistical analysis

We first conducted descriptive analyses in which categorical variables and quantitative variables were presented as frequencies and percentages or as the mean (SD). Population characteristics by sleep pattern categories were compared by Pearson’s Chi-squared test or one-way ANOVA, as appropriate.

In the next phase of analysis, the linear regression model (LM) and generalized linear model (GLM) in *STATA* (version 17) were employed to characterize how sleep and PA related to each of the six brain imaging metrics and four cognitive outcomes, after fully adjusting for covariates, including sex, age, ethnicity, education level, TDI, smoking status, alcohol intake frequency, BMI, WHR, hypertension, diabetes, and lipidaemias. To correct adequately for effects of sex and age, we also covaried the age×sex interaction. Before applying a regression model, we log-transformed the summed MET minutes, and the WMH, DWMH, and PWMH volumes to correct for their heavily skewed distributions. Besides, brain MRI parameters were centered and standardized. LM was used for all imaging phenotypes, while GLM with Poisson error structure and log link function was applied for counting (e.g., symbol digit substitution) cognitive phenotypes. Using the above method, we first tested associations between sex, age, and their interaction with all exposures and outcomes. Then, the independent and joint effects of sleep behaviors and IPAQ groups on each brain and cognitive imaging phenotypes were examined, taking healthy sleep patterns, normal sleep duration, and moderate PA as the references. Subsequently, to determine whether a certain level of PA could attenuate brain structural changes and declining cognition arising in relation to unhealthy sleep behaviors, we investigated the interactive associations of sleep behaviors and PA levels with neuroimaging and cognitive outcomes. Moreover, we included total intracranial volume as a confounder for sensitivity analyses when conducting tests of brain-level associations. We excluded participants who were regularly involved in heavy physical jobs and night shifts for sensitivity analyses. Finally, we employed a linear mixed model to examine the longitudinal relationship of sleep duration and physical activity on brain structure change, and the model excluded participants with a one-year follow-up (*n* = 98).We report relevant summary statistics and two-tailed *P* values in all of the above analyses and corrected for multiple comparisons to control for false discovery rate (FDR) [25] using the ‘p.adjust*’* function in *R* (version 4.1.2). Corrected *p*-values below 0.05 were considered significant.

## 3 Results

### 3.1 Baseline characteristics of the study participants

The demographic characteristics of the study participants by categories of sleep patterns are detailed in Table 1. The 42,065 included participants were of the mean (SD) age 64.22 (7.77) years, of whom 20,268 (48.18%) were male. The majority (50.57%) of the participants reported healthy sleep, with 44.44% having intermediate sleep and 4.99% having poor sleep. Participants who were women, and those with higher education, lower BMI and WHR, never smoking, generally fewer diagnoses of common vascular risk factors, and higher PA had healthier sleep patterns. Differences in the brain MRI and cognition measures among the three groups suggested that the healthy sleep pattern group had better preservation of brain structure and cognitive function. The modeling effects of age, sex, and age × sex on each sleep behavior, PA levels, brain MRI parameters, and cognitive phenotypes indicated that age and sex had significant effects on all indices (Table S6). Besides, the correlations among the studied variables were generally significant (Table S7).

**Table 1:** **Characteristics of the study participants by categories of sleep patterns**.

Characteristics	Sleep patterns					*P* value
	Nsum	Total	Healthy	Intermediate	Poor	
**Demographics**	**N**					
Age, years [M (SD)]	42, 065	64.22 (7.77)	64.06 (7.76)	64.44 (7.77)	63.85 (7.78)	<0.001
Sex, male [n (%)]	42, 065	20,268 (48.18)	9766 (45.91)	9403 (50.29)	1099 (52.38)	<0.001
Ethnicity, white [*n* (%)]	42, 065	40,825 (97.05)	20,737 (97.49)	18,092 (96.77)	1996 (95.14)	<0.001
Education level [*n* (%)]	42, 065					<0.001
No qualification		2728 (6.49)	1229 (5.78)	1357 (7.26)	142 (6.77)	
Low		9162 (21.73)	4375 (20.57)	4242 (22.69)	545 (25.98)	
Medium		7706 (18.32)	3826 (17.99)	3469 (18.55)	411 (19.59)	
High		22,469 (53.41)	11,841 (55.67)	9628 (51.50)	1000 (47.66)	
Townsend deprivation index [M (SD)]	42, 065	-1.95 (2.68)	-2.01 (2.65)	-1.92 (2.70)	-1.95 (2.68)	<0.001
**Vascular risk factors**						
Smoking status [*n* (%)]	42, 065					<0.001
Never		26,214 (62.32)	13,747 (64.63)	11,284 (60.36)	1183 (56.39)	
Previous		14,416 (34.27)	6939 (32.62)	6663 (35.64)	814 (38.80)	
Current		1435 (3.41)	585 (2.75)	749 (4.01)	101 (4.81)	
Alcohol intake frequency [*n* (%)]	42, 065					<0.001
Never		6945 (16.51)	3394 (15.96)	3130 (16.74)	421 (20.07)	
Sometimes		15,950 (37.92)	8203 (38.56)	6995 (37.41)	752 (35.84)	
Often		19,170 (45.57)	9674 (45.48)	8571 (45.84)	925 (44.09)	
Hypertension [*n* (%)]	42, 065	12,542 (29.82)	5550 (26.09)	6164 (32.97)	828 (39.47)	<0.001
Diabetes [*n* (%)]	42, 065	2260 (5.37)	868 (4.08)	1197 (6.40)	195 (9.29)	<0.001
Lipidaemias [*n* (%)]	42, 065	9295 (22.10)	4165 (19.58)	4535 (24.26)	595 (28.36)	<0.001
BMI, kg/m^2^ [M (SD)]	42, 065	26.59 (4.47)	25.97 (4.14)	27.07 (4.60)	28.60 (5.29)	<0.001
WHR [M (SD)]	42, 065	0.88 (0.09)	0.87 (0.09)	0.89 (0.09)	0.90 (0.09)	<0.001
**Sleep behaviors**						
Sleep duration [*n* (%)]	42, 065					<0.001
Short		10,005(23.78)	1432 (6.73)	7096 (37.95)	1477 (70.40)	
Normal		28,930 (68.77)	19,132 (89.94)	9511 (50.87)	287 (13.68)	
Long		3130 (7.44)	707 (3.32)	2089 (11.17)	334 (15.92)	
Chronotype, late [*n* (%)]	42, 065	27,745 (65.32)	17,256 (82.39)	9497 (50.80)	452 (21.54)	<0.001
Insomnia [*n* (%)]	42, 065	13,464 (32.01)	2357 (11.08)	9294 (49.71)	1813 (86.42)	<0.001
Snoring [*n* (%)]	42, 065	15,269 (36.30)	3915 (18.41)	9576 (51.22)	1778 (84.75)	<0.001
Narcolepsy [*n* (%)]	42, 065	9875 (23.48)	1717 (8.07)	6567 (35.13)	1591 (75.83)	<0.001
**Physical activity**						
Summed MET minutes/week [M (SD)]	42, 065	2698.00 (2415.18)	2882.24 (2452.03)	2535.97 (2359.01)	2273.96 (2377.48)	<0.001
IPAQ activity group [*n* (%)]	42, 065					<0.001
Low		4567 (10.86)	1781 (8.37)	2418 (12.93)	368 (17.54)	
Moderate		19,091 (45.38)	9329 (43.86)	8734 (46.72)	1028 (49.08)	
High		18,407 (43.76)	10,161 (47.77)	7544 (40.35)	702 (33.46)	
**Brain MRI parameters**						
Grey-White matter volume, mm^3^ [M (SD)]	33, 301	1,494,511 (73,236.94)	1,495,673 (73,107.05)	1,493,182.00 (73,249.43)	1,494,364.00 (74,313.09)	0.011
Grey matter volume, mm^3^ [M (SD)]	33, 298	792,699.70 (47,992.56)	794,167.70 (47,665.84)	791,245.20 (48,295.22)	790,476.90 (48,201.38)	<0.001
Peri-Corti grey volume, mm^3^ [M (SD)]	33, 299	617,976.80 (40,879.21)	618,969.70 (40,643.19)	616,964.60 (41,136.71)	616,728.50 (40,775.97)	<0.001
WMH volume, mm^3^ [M (SD)]	31, 926	4494.89 (4884.74)	4314.72 (4738.60)	4663.89 (5021.80)	4856.41 (5062.85)	<0.001
PWMH volume, mm^3^ [M (SD)]	34, 700	3673.39 (3756.88)	3537.11 (3663.30)	3808.56 (3857.90)	3870.68 (3742.18)	<0.001
DWMH volume, mm^3^ [M (SD)]	34, 649	861.60 (1391.19)	825.65 (1360.23)	895.77 (1421.83)	926.76 (1420.02)	<0.001
**Cognition parameters**						
Matrix pattern [M (SD)]	28, 412	7.94 (2.13)	7.98 (2.12)	7.91 (2.15)	7.76 (2.15)	<0.001
Tower rearranging [M (SD)]	28, 194	9.83 (3.24)	9.86 (3.22)	9.81 (3.26)	9.76 (3.30)	0.328
Symbol digit attempted [M (SD)]	22, 228	19.56 (4.96)	19.73 (4.97)	19.40 (4.98)	19.09 (4.64)	<0.001
Symbol digit correctly [M (SD)]	22, 228	18.77 (5.33)	18.98 (5.29)	18.60 (5.37)	18.23 (5.17)	<0.001

Brain MRI volumes are normalized for head size.

Abbreviations: M (SD), mean (standard deviation); BMI, body mass index; WHR, waist-hip ratio; MET, metabolic equivalent; IPAQ, International Physical Activity Questionnaire; SBP, systolic blood pressure; DBP, diastolic blood pressure; Peri-Corti grey volume, Peripheral cortical grey matter volume; WMH, white matter hyperintensity; PWMH, peri‑ventricular white matter hyperintensities; DWMH, deep white matter hyperintensities.

### 3.2 Associations of sleep behaviors and PA levels with brain imaging parameters

**Fig. 2:**
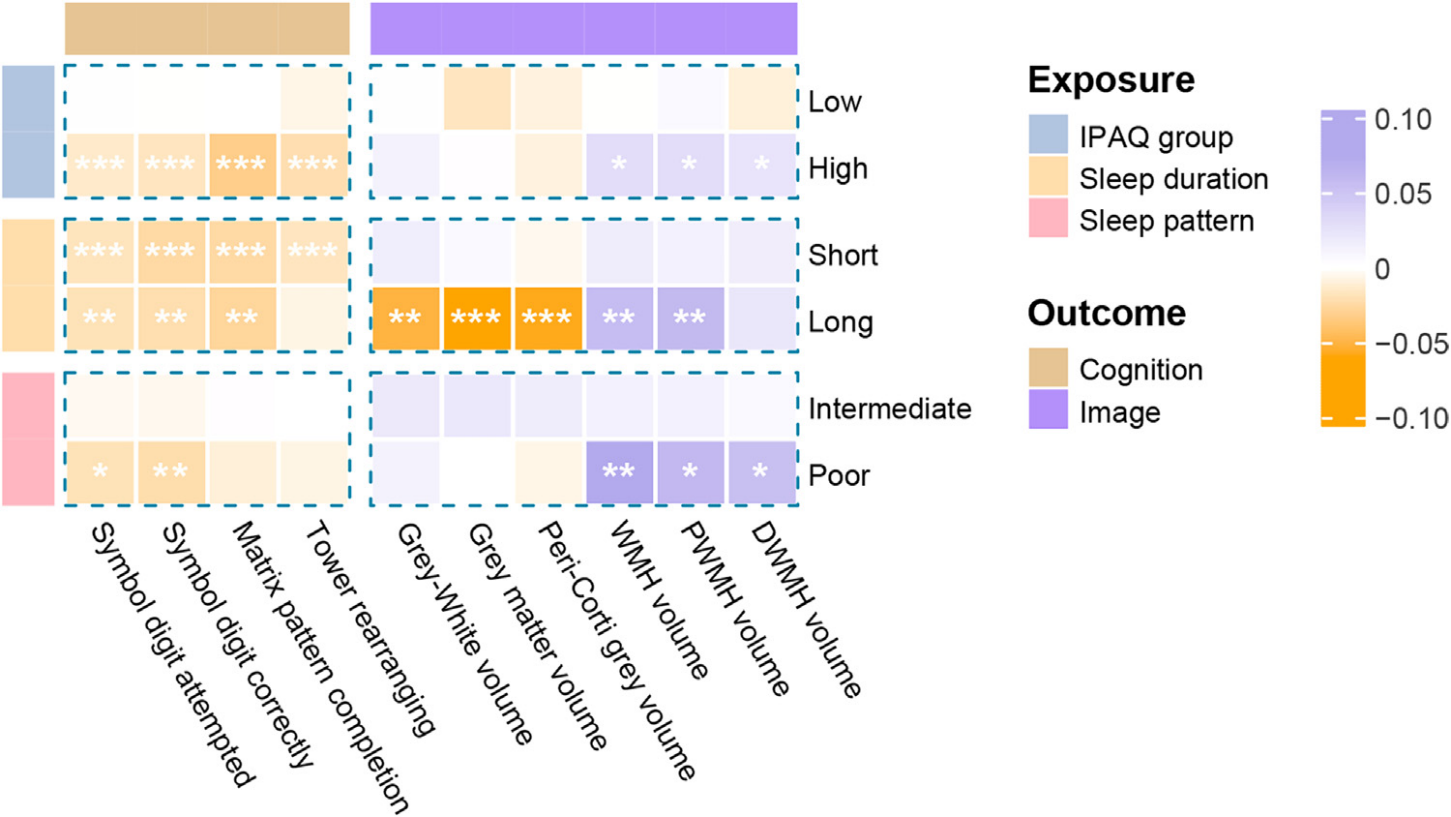
**Independent associations of sleep behaviors and PA levels with brain imaging parameters and cognition performance.** This figure shows that long sleep duration was independently associated with brain structure damage, and both inappropriate sleep duration and high PA level negatively impacted cognitive function. Abbreviations: IPAQ, International Physical Activity Questionnaire; Peri-Corti grey volume, Peripheral cortical grey matter volume; WMH, white matter hyperintensity; PWMH, peri‑ventricular white matter hyperintensities; DWMH, deep white matter hyperintensities. All results were based on models adjusting for sex, age, ethnicity, education level, Townsend deprivation index, smoking status, alcohol intake frequency, body mass index, waist-hip ratio, hypertension, diabetes and lipidaemias. The star (*) denotes the *P* values after multiple tests with the false discovery rate method. *P < 0.05; ^⁎⁎^*P* < 0.01; ^⁎⁎⁎^*P* < 0.001.

In independent analyses (Fig. 2 and Table S8), our analyses of the effects of sleep duration on brain MRI measures revealed that long sleep duration was independently associated with smaller global brain (*β* = -0.052, *P*_FDR_ = 0.003), grey matter (*β* = -0.061, *P*_FDR_ = 1.1 × 10^-^^4^) and peripheral cortical grey (*β* = -0.059, *P*_FDR_ = 2.9 × 10^-^^4^) volume and greater WMH (*β* = 0.057, *P*_FDR_ = 0.003) and PWMH (*β* =0.059, *P*_FDR_ = 0.001) volume, taking normal sleep duration as reference. Individuals with poor sleep patterns had higher WMH, PWMH, and DWMH burden (*β* range 0.054 to 0.074, *P*_FDR_ < 0.050) than healthy sleepers. Compared to the moderate PA level, a high level of PA was associated with greater WMH, PWMH, and DWMH volumes (*β* range 0.024 to 0.029, *P*_FDR_ < 0.050).

**Fig. 3:**
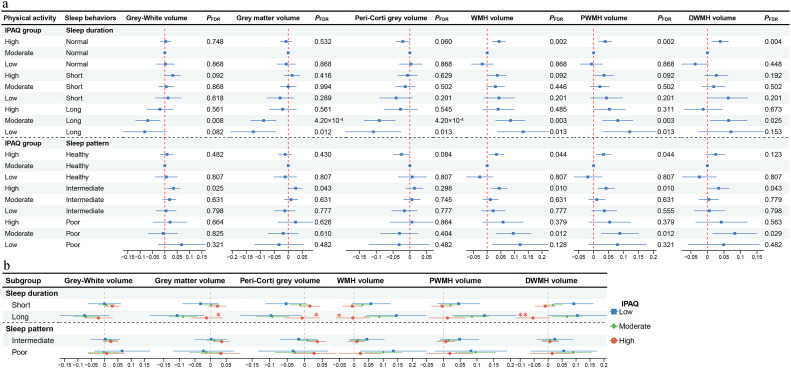
**Associations of sleep behaviors and PA levels with brain imaging parameters.** (a) Long sleep duration with low PA levels significantly exhibits the greatest deleterious effect on the brain MRI parameters. (b) High PA levels mitigate against the detrimental effect of brain structural damage caused by long sleep duration. Abbreviations: IPAQ, International Physical Activity Questionnaire; Peri-Corti grey volume, Peripheral cortical grey matter volume; WMH, white matter hyperintensity; PWMH, peri‑ventricular white matter hyperintensities; DWMH, deep white matter hyperintensities. All results were based on models adjusting for sex, age, ethnicity, education level, Townsend deprivation index, smoking status, alcohol intake frequency, body mass index, waist-hip ratio, hypertension, diabetes and lipidaemias. *P*_FDR_ was the value after multiple tests with the false discovery rate method. The red star (*) denotes *P* values of interaction between the group of high and moderate PA levels. *P < 0.05; ^⁎⁎^*P* < 0.01; ^⁎⁎⁎^*P* < 0.001.

The results of joint associations of sleep behaviors and PA levels with brain imaging phenotypes are shown in Fig. 3a and Table S9. Compared to those with normal sleep duration and a moderate level of PA, the greatest deleterious effect on the brain MRI parameters (such as GWV, *β* = -0.125, *P*_FDR_ = 0.012; WMH, *β* = 0.131, *P*_FDR_ = 0.013) was observed among individuals with long sleep duration and low level of PA, followed by long sleepers with moderate PA level (such as GWV, *β* = -0.088, *P*_FDR_ = 4.2 × 10^-^^4^; WMH, *β* = 0.085, *P*_FDR_ = 0.003). The greatest white matter injury was present in the group with poor sleep patterns and a low/moderate level of PA (WMH, *β*_low_ = 0.120, *P*_FDR_ = 0.128; PWMH, *β*_moderate_ = 0.088, *P*_FDR_ = 0.012; DWMH, *β*_moderate_ = 0.083, *P*_FDR_ = 0.029) compared with the reference group.

When categorized by the PA levels, long sleep duration was related to brain atrophy (such as GWV, *β*_low_ = -0.107, *P*_FDR_ = 0.031; *β*_moderate_ = -0.088, *P*_FDR_ = 4.5 × 10^-^^4^) and increased WMH/PWMH/DWMH burden (such as WMH, *β*_low_ = 0.145, *P*_FDR_ = 0.029; *β*_moderate_ = 0.087, *P*_FDR_ = 0.002) compared to normal sleep duration under the low/moderate PA level. However, effects on the above brain structural indices in association with long sleep duration among those with a high PA level were similar to findings in the reference group (GWV, *β*_high_ =-0.013, *P*_FDR_ = 0.891, *P* for interaction = 0.024; WMH, *β*_high_ = -0.004, *P*_FDR_ = 0.891, *P* for interaction = 0.027), indicating that high PA level may mitigate against the brain structural changes in association with increased sleep duration. Similarly, among those engaging in moderate PA, there was no significant difference in brain volumes between intermediate sleepers and healthy sleepers, but the intermediate sleep pattern was associated with greater grey (*β* = 0.036, *P*_FDR_ = 0.014) and peripheral cortical grey matter volume (*β* = 0.036, *P*_FDR_ = 0.014) compared to the group with healthy sleep pattern under a high level of PA. Moreover, under moderate PA levels, poor sleepers exhibited greater WMH/PWMH/DWMH burden (WMH, *β* = 0.101, *P*_FDR_ = 0.006) compared with healthy sleepers, but the adverse effect was not significant among those with high levels of PA (WMH, *β* = 0.022, *P*_FDR_ = 0.813). Although the interacting associations of sleep patterns and PA levels with brain structural changes were statistically insignificant, the findings still suggest that the detrimental association of unhealthy sleep patterns was smaller among individuals with high levels of PA (Fig. 3b and Table S10).

Sensitivity analysis suggested that all of the above results remained significant after adjusting for total cranial volume (Tables S11, S12, S13) and excluding heavy laborers and night shift workers (Tables S17, S18, S19). The longitudinal analysis partly validated our findings, revealing that a high level of PA appeared to offset the detrimental effects of extended sleep on deep white matter changes (*P* for interaction = 0.045) (Tables S23, S24, S25).

### 3.3 Associations of sleep behaviors and PA levels with cognition performance

For cognition phenotypes, taking normal sleep duration as a reference, both short and long sleep duration negatively impacted cognitive function in most cognition phenotypes (such as symbol digit attempted, *β*_short_ = -0.018, *P*_FDR_ < 1.0 × 10^-^^6^; *β*_long_ = -0.020, *P*_FDR_ = 0.001), except for tower rearranging completion. Poor sleep patterns negatively impacted the symbol digit substitution test (*β* = -0.023, *P*_FDR_ = 0.009). Additionally, individuals with a high level of PA had worse cognitive function across all phenotypes than those with a moderate level of PA (Fig. 2 and Table S14).

**Fig. 4:**
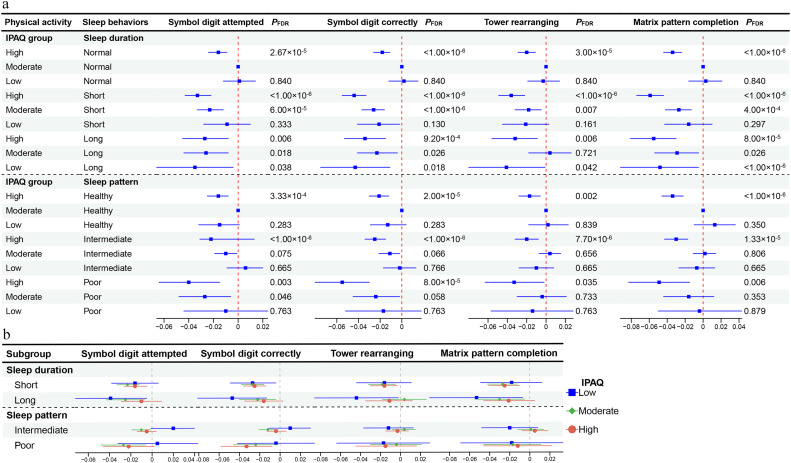
**Associations of sleep behaviors and PA levels with cognition performance.** (a) Long sleep duration coupled with low level of PA is significantly associated with poor cognitive performance, as well as the combination of unhealthy sleep behaviors with high level of PA. (b) Higher PA level is not sufficient to attenuate the cognitive decline association with long sleep duration. Abbreviations: IPAQ, international physical activity questionnaire. All results were based on models adjusting for sex, age, ethnicity, education level, Townsend deprivation index, smoking status, alcohol intake frequency, body mass index, waist-hip ratio, hypertension, diabetes and lipidaemias. *P*_FDR_ was the value after multiple tests with the false discovery rate method.

The results of joint analyses of sleep behaviors and PA levels with cognition phenotypes are displayed in Fig. 4a and Table S15. When taking individuals with normal sleep duration and a moderate level of PA as a reference, the combination of long sleep duration with a low level of PA was associated with the worst cognitive performance (such as symbol digit correctly, *β* = -0.035, *P*_FDR_ = 0.038), followed by the combination of short sleep with a high level of PA (such as symbol digit correctly, *β* = -0.033, *P*_FDR_ < 1.0 × 10^-^^6^). Compared to those with healthy sleep patterns in conjunction with moderate levels of PA, we found the group with poor sleep patterns and high levels of PA performed worst on most cognitive phenotypes (such as matrix pattern completion, *β* = -0.049, *P*_FDR_ = 0.006).

Associations of sleep behaviors and cognition stratified by PA levels are shown in Fig. 4b and Table S16. Under the low level of PA, we found that long sleep duration exhibited a negative impact on cognitive phenotypes compared to normal sleep duration. Even though this effect was smaller or insignificant between long and normal sleepers engaging the moderate or high level of PA, i.e., in scores of matrix pattern completion (*β*_low_ = -0.053, *P*_FDR_ = 0.033; *β*_moderate_ = -0.030, *P*_FDR_ = 0.025; *β*_high_ = -0.021, *P*_FDR_ = 0.239), the interactions were insignificant, which suggests that moderate or high PA might not attenuate the cognitive decline arising in association with long sleep duration. Nevertheless, when categorized by PA levels, there was no significant difference between short sleepers and normal sleepers in the effects on cognitive performance at any level of PA, nor did this differ between those with poor and healthy sleep patterns.

To avoid the influence of heavy physical labor work and shift work on cognitive function, we conducted a sensitivity analysis by excluding heavy laborers and night shift workers. The findings remained consistent with our main analysis results, further supporting the robustness of our conclusions (Tables S20, S21, S22).

## 4 Discussion

This is the first study in a large cohort to indicate that sleep behaviors and PA levels have significantly additive and interactive effects on brain MRI measures and cognitive performance in aging. We found that unhealthy sleep behaviors and high PA levels independently exhibited negative impacts on brain structure and cognitive function. When combining sleep behaviors with PA levels, the greatest brain structural changes and the worst cognitive performance were present in the group with long sleep duration (exceeding 8 h per night) and low levels of PA. The most striking finding was that high levels of PA significantly mitigate the adverse effects of excessive sleep duration on brain structure. These findings support the hypothesis that participation in moderate or high levels of PA could maintain brain health for long sleepers.

Sleep duration is a key indicator of brain and cognitive health. Our findings are consistent with previous studies suggesting that insufficient and excessive sleep duration were both associated with worse cognitive performance [26], while prolonged sleep duration was linked with WMH burden [27] and smaller total brain and grey matter volumes [28]. However, those studies overlooked the impact of other sleep behaviors on sleep duration [21]. Given these cross-correlations of sleep-related factors, we calculated a healthy sleep score composed of five sleep behaviors, thus providing a framework for defining a healthy sleep pattern in our study. To date, some studies have similarly combined five sleep behaviors in a composite healthy sleep score to explore the relationship of sleep patterns with disease [21] and brain image [29]. The present findings are in accord with a previous study suggesting an association between unhealthy sleep patterns with high WMH burden [29]. Moreover, our analysis expands on the relationship between sleep patterns and cognition, finding that those with poor sleep performed worse specifically in the symbol digit test. This discovery underscores the significance of sleep duration over sleep patterns in preserving brain and cognitive health.

PA is recognized by WHO guidelines as a key focus for cognitive health [30]. To date, the effect of physical activity on cognitive performance remains a subject of ongoing debate. Research indicates that the relationship between exercise intensity, type, and duration, and its influence on cognitive function is complex and not always clear-cut. While some physiological mechanisms suggest that PA may boost cognition, other studies propose that it could potentially have a negative impact on cognitive performance, especially in the context of strenuous exercise. Certain studies have indicated that prolonged intense exercise can lead to the accumulation of high levels of reactive oxygen species, which may cause oxidative damage and elevate neuronal mortality [31]. A population-based cohort study indicated that brain health in young and middle-aged adults may particularly profit from additional high-intensity physical activities, while additional light-intensity activities may suffice to maintain brain health in older adults [8]. Additionally, a study involving older adults suggested that high-intensity interval exercise could have a detrimental effect on the hippocampus and overall cognitive performance [32]. Our study involving middle-aged and older participants, with a mean age of 64.2 years, gave findings that were consistent with previous studies. Furthermore, another Mendelian randomization study also indicated that prolonged moderate to vigorous physical activity is associated with a decline in cognitive performance [33], in accordance with present results. Despite concerns about its potential negative impact on cognitive performance, the overall health benefits of physical activity justify its promotion across the general population. However, we recommended caution about prolonged vigorous PA in the elderly, to minimize potential adverse effects on cognitive function. Future research should investigate the effects of various exercise types and intensities at the genetic level to identify the most beneficial forms of exercise for enhancing cognitive performance and overall health while minimizing the risk of cognitive decline.

Concerning the combined impact of sleep duration and PA, few studies have explored their interactive effect on brain health and cognitive function. Our research addressed this gap and revealed that excessive sleep duration exhibited the most significant brain damage in individuals engaging in low levels of PA, but a high PA level significantly mitigates the adverse effects of long sleep duration on brain structure. However, participation in a high level of PA seemed insufficient to alleviate the cognitive decline associated with excessive sleep. Several studies have attempted to explore the additive effects of sleep duration and PA on mortality risk [34] and cognitive function [35]. Indeed, this phenomenon was corroborated by a longitudinal study, which observed a more rapid decline in individuals with higher PA and short sleep aged 50 to 60 years over a 10-year follow-up period [35]. In general, consistent with previous literature findings, present results support the need to encourage adequate PA levels according to individual sleep behaviors for optimizing brain and cognitive health.

We found that higher PA levels alleviated signs of brain structural changes among long sleepers as compared with normal sleepers. We propose several possible mechanisms to explain this result. First, sleep may promote white matter integrity through glymphatic clearance of neurotoxic metabolites and damaged neuron DNA, the promotion of oligodendrocyte precursor cells for myelin formation, and maintaining homeostasis of synaptic connections [36]. Prolonged sleep duration may disrupt that homeostasis, resulting in brain atrophy or white matter changes. Furthermore, increasing sleep duration may represent an early stage of somatic disease [37]. Conversely, the multiple health benefits of PA include improved cardiorespiratory fitness [38], inhibition of inflammatory responses [39], and improved insulin sensitivity [40], each of which factors may compensate for the detrimental association of long sleep duration with brain health.

We availed ourselves of the large sample of standardized imaging data in the UK Biobank and considered a net metric of five sleep behaviors in conjunction with PA levels. More importantly, PA and sleep duration are both time-dependent behaviors that are amenable to interventions. We suppose that concurrent targeting of PA and sleep duration recommendations may impart super-additive greater benefits in maintaining brain health. Given the extremes of excessive sleep, sufficient PA offers a practical way to partly offset the negative effects of unhealthy sleep on brain health.

However, this study had some potential limitations. First, although we endeavored to attain due consideration of confounding factors, we could not adjust for some potential residual covariates, such as the use of sleeping pills. Second, sleep behaviors and PA were self-reported, nevertheless, the evidence suggested that the conclusions drawn from the self-reported data align with objectively measured data [35,41]. Third, the sample from the UK Biobank consists mainly of individuals of European descent, which may limit the generalizability of the current findings. Finally, the statistical significance of our longitudinal analysis is not powerful enough, potentially due to the relatively short follow-up period (mean 2.6 years) and the small sample size of certain groups (*n* = 47), which made it challenging to fully assess the effects of baseline sleep and physical activity on brain structure changes. Thus, future studies with longer follow-up durations and larger sample sizes are required to further validate our findings.

## 5 Conclusion

In summary, this study showed that poor sleep behavior, especially long sleep duration, was associated with greater brain structural damage and worse cognitive function in the elderly population, but a high level of PA may mitigate this detrimental effect. Our findings highlight that the promotions targeting both sleep duration and PA may be more effective in preventing or delaying brain structure damage and cognitive decline among the middle-aged population than solely targeting either behavior. Both sleep habits and PA levels should be addressed to maximize benefits for long-term maintenance of brain and cognitive health.

## Declaration of competing interests

The authors declare that they have no conflicts of interest in this work.

## CRediT authorship contribution statement

**Yuanyuan Fang:** Data curation, Formal analysis, Software, Visualization, Writing – original draft. **Chang Cheng:** Data curation, Visualization, Writing – original draft. **Deng Ning:** Data curation, Visualization, Writing – original draft. **Yao Yao:** Data curation. **Lusen Ran:** Investigation. **Yuqin He:** Investigation. **Hao Huang:** Investigation. **Di Yao:** Investigation. **Yanzhu Huang:** Supervision, Validation. **Dengji Pan:** Resources. **Wei Wang:** Conceptualization, Resources, Writing – review & editing. **Wenhua Liu:** Formal analysis, Methodology, Project administration, Software, Supervision, Validation, Writing – review & editing. **Minghuan Wang:** Conceptualization, Funding acquisition, Resources, Supervision, Validation, Writing – review & editing.

## Appendix Supplementary materials

Image, application 1
